# Consecutive sexual maturation observed in a rock shell population in the vicinity of the Fukushima Daiichi Nuclear Power Plant, Japan

**DOI:** 10.1038/s41598-020-80686-3

**Published:** 2021-01-12

**Authors:** Toshihiro Horiguchi, Kayoko Kawamura, Yasuhiko Ohta

**Affiliations:** 1 National Institute for Environmental Studies, Tsukuba, Ibaraki 305-8506 Japan; 2 Faculty of Agriculture, Tottori University, Tottori, Tottori 680-8553 Japan

**Keywords:** Environmental impact, Ecological epidemiology

## Abstract

In 2012, after the accident at the Fukushima Daiichi Nuclear Power Plant (FDNPP) that followed the Tohoku earthquake and tsunami in March 2011, no rock shell (*Thais clavigera*; currently recognized as *Reishia clavigera*; Gastropoda, Neogastropoda, Muricidae) specimens were found near the plant from Hirono to Futaba Beach (a distance of approximately 30 km). In July 2016, however, rock shells were again found to inhabit the area. From April 2017 to May 2019, we collected rock shell specimens monthly at two sites near the FDNPP (Okuma and Tomioka) and at a reference site ~ 120 km south of the FDNPP (Hiraiso). We examined the gonads of the specimens histologically to evaluate their reproductive cycle and sexual maturation. The gonads of the rock shells collected at Okuma, ~ 1 km south of the FDNPP, exhibited consecutive sexual maturation during the 2 years from April 2017 to May 2019, whereas sexual maturation of the gonads of specimens collected at Hiraiso was observed only in summer. The consecutive sexual maturation of the gonads of the specimens collected at Okuma might not represent a temporary phenomenon but rather a site-specific phenotype, possibly caused by specific environmental factors near the FDNPP.

## Introduction

The 2011 Tohoku earthquake (M_w_ 9.0) on 11 March 2011 generated a tsunami that caused meltdown of three nuclear reactors at the Fukushima Daiichi Nuclear Power Plant (FDNPP), owned by the Tokyo Electric Power Company. Hydrogen explosions in the reactor buildings caused the emission of hundreds of petabecquerels (PBq) of radionuclides to the environment^1^. Nevertheless, the estimated amount of radionuclide leakage from the FDNPP is only about one-tenth the total amount released by the 1986 Chernobyl Nuclear Power Plant disaster in Ukraine, which has been estimated as 5300 PBq, excluding noble gases^1^. Releases of FDNPP-derived radionuclides to the environment have occurred via four major pathways^2^: 1) Earliest and largest was the initial venting and explosive release of gases and volatile radionuclides to the atmosphere. 2) Subsequently, contaminated material, including radionuclides, was leaked directly from the reactors to the sea during emergency cooling efforts. Finally, releases of radionuclides to the sea via 3) groundwater discharge and 4) river runoff are ongoing. The amounts released via groundwater and river discharge are much smaller than the initial atmospheric fallout and the direct leakage to the sea. Although transport model simulations have indicated that more than 80% of the atmospheric fallout during the 2011 disaster was onto the ocean surface, and that deposition was highest onto coastal waters near the FDNPP, this evaluation was not confirmed by direct observations of atmospheric fallout over the ocean^2^. Several studies have estimated that total deposition from the atmosphere onto the ocean surface was 5–15 PBq, and direct leakage of ^137^Cs from the FDNPP into the sea was 3–6 PBq^3–12^. Less is known about the amounts of other chemical substances leaked from the FDNPP to the environment (especially the marine environment) following the nuclear disaster, although there is limited information about boric acid and hydrazine^13^.

Despite the large number of studies that have estimated the amount of radionuclide leakage from the FDNPP to the environment and that have examined the behavior and fate of leaked radionuclides in the environment, little is known about the ecological effects of radionuclides generally, and those derived from the nuclear disaster in particular, on wildlife^13–16^. However, some data on the activity concentrations of radionuclides (i.e., gamma emitters) in aquatic organisms (fishes and shellfishes) have been collected, mainly for regulatory purposes^17–20^.

Starting in December 2011, following the FDNPP accident in March 2011, we investigated the intertidal zones along the coastline of eastern Japan to evaluate possible ecological effects of harmful substances leaked from the FDNPP into the sea on marine organisms inhabiting areas close to the FDNPP. We found that in 2012 the number of intertidal species decreased significantly with decreasing distance from the power plant. In particular, we were able to collect no rock shell (*Thais clavigera*) specimens along the coast near the plant from Hirono to Futaba Beach, a distance of approximately 30 km, even though we were able to collect rock shell specimens from many other sites struck by the tsunami^13^. Furthermore, quantitative surveys in 2013 showed that species richness and population densities of sessile invertebrates, especially Arthropoda, in intertidal zones were much lower at sites near the FDNPP or within several kilometers southward than at other sites, and they were also lower in 2013 than in 1995. These findings strongly suggest that the intertidal biota around the power plant was affected by the nuclear accident^13^.

According to the results of quantitative quadrat surveys of sessile invertebrates conducted from 2014 to 2019 at seven intertidal sites in Ibaraki, Fukushima, and Miyagi prefectures, including sites near the FDNPP, species richness and population densities in intertidal zones near the FDNPP did not start to increase again until at least 4–5 years after the 2011 disaster^14^. In April, July, August, and September from 2012 to 2017, we also monitored the population density and spawning behavior of rock shells at sites near the FDNPP. Although in July 2016, rock shells were again found to be inhabiting the area where no rock shells were found in 2012, rock shell population densities and reproductive performance near the FDNPP remained lower in 2017 than before the accident. Thus, sessile invertebrate larval recruitment from remote areas to the intertidal zones near the FDNPP was not clearly observed until 2016 at the earliest. These findings suggest that environmental factors inhibited invertebrate reproduction, recruitment, or both in the intertidal zones near the FDNPP^14^.

**Figure 1:**
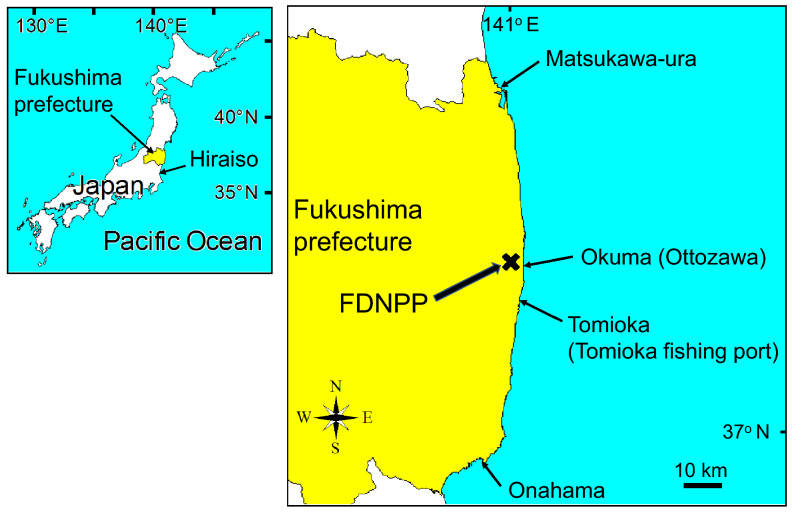
Sampling sites of the rock shell (*Thais clavigera* and *Thais bronni*) between April 2017 and May 2019. Two sites were near the Fukushima Daiichi Nuclear Power Plant (FDNPP) in Fukushima prefecture: Ottozawa, Okuma town, and Tomioka fishing port, Tomioka town are approximately 1 km and 10 km south of the FDNPP, respectively. The reference site, Hiraiso, Hitachi-naka city, Ibaraki prefecture, is approximately 120 km south of the FDNPP. Water temperature was observed at Onahama, Iwaki city, and at Matsukawa-ura, Soma city, by the Fukushima Prefectural Research Institute of Fisheries Resources. *The maps used in this figure are based on a map of Japan available at http://www5b.biglobe.ne.jp/~t-kamada/CBuilder/mapmap.htm and have been modified by the authors.

Besides the recovery or increase in rock shell populations at sites near the FDNPP, normal reproduction, in terms of sexual maturation, copulation, egg-laying, hatching, survival, and growth (including metamorphosis and settlement) in the early life history stages and recruitment to populations, is also important to maintain rock shell populations, including at sites near the FDNPP. Therefore, to investigate whether normal reproduction was occurring, we collected rock shell (*T. clavigera* and *Thais bronni*) specimens monthly from April 2017 to May 2019 at two sites in Fukushima prefecture near the FDNPP (Ottozawa, Okuma town, ca. 1 km south of the FDNPP, and Tomioka fishing port, Tomioka town, ca. 10 km south of the FDNPP) and at a reference site in Ibaraki prefecture (Hiraiso, Hitachi-naka city, ca. 120 km south of the FDNPP) (Fig. 1 and Supplementary Table S1). We then examined their gonads histologically to evaluate reproductive cycles and sexual maturation in these specimens. Our results revealed a strange phenomenon—consecutive sexual maturation—in a rock shell population in the vicinity of the FDNPP.

## Results

### Reproductive cycles and sexual maturation in female *T. clavigera* and *T. bronni*

**Figure 2:**
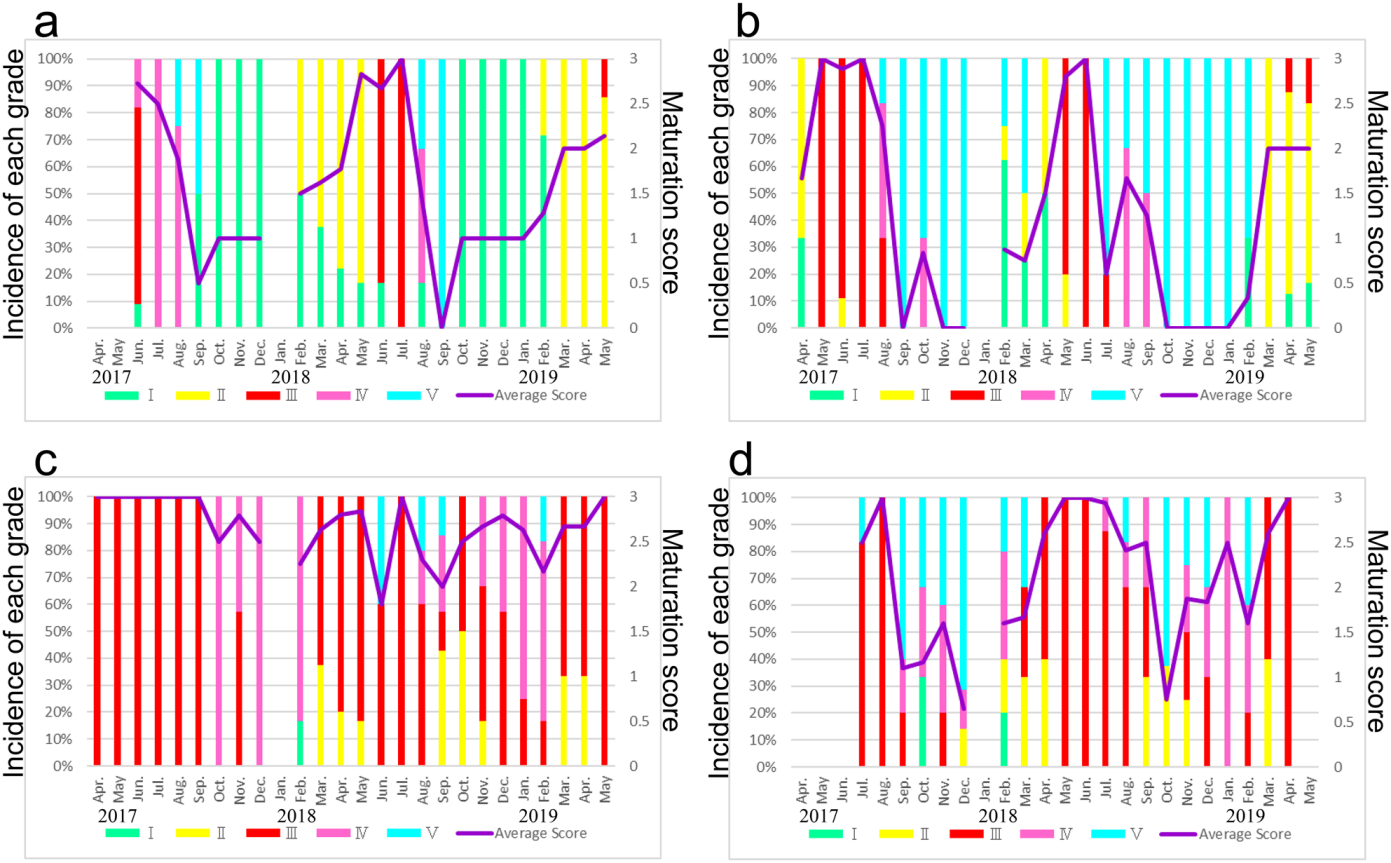
Time series of maturation scores (purple line) and developmental grades (colored vertical bars) of female *Thais clavigera* and *Thais bronni* specimens collected at (**a**) Hiraiso (*T. clavigera*), (**b**) Tomioka (*T. clavigera*), (**c**) Okuma (*T. clavigera*), and (**d**) Okuma (*T. bronni*).

Female reproductive cycles were recognized in temporal changes in the monthly average sexual maturation scores of ovaries from *T. clavigera* and *T. bronni* specimens collected at each site (Fig. 2). Ovaries of developmental grades III and IV (corresponding to maturation scores of 3 and 2.5, respectively) in *T. clavigera* and *T. bronni* were recognized as sexually mature (Fig. 2 and Table 1).

**Table 1:** Stages, developmental grades, and maturation scores of ovaries of *Thais clavigera* and *Thais bronni*.

Stage	Developmental grade	Characteristics of ovarian tissue	Primary cells observed in the ovary	Maturation score
1. Recovery stage	I	Ovarian tissue is thin. Connective tissues, hemocytes, yellow-white granules, and rarely yolk globules are observed	Oogonia and oocytes are at an early stage (> 80%)	1
2. Active stage	II	Ovarian tissue is becoming thicker. Oogonia and different stages of oocytes are observed, with accumulating yolk granules and globules. Connective tissues and hemocytes are rarely observed	Oogonia and oocytes are at different stages	2
3. Mature stage	III	Ovarian tissue is thick (approximately > 50% of the cross section of ovary and digestive gland). Ovarian follicles are filled with yolk globules. The outline of mature oocytes is unclear. No connective tissue is observed	Mature oocytes (> 60%) and a few oogonia	3
	IV	Ovarian tissue is thick (approximately > 50% of the cross section of ovary and digestive gland), and the ovarian follicle is filled with yolk globules. The outline of mature oocytes is unclear. However, an aperture/void is observed within the ovarian follicle. Connective tissue and hemocytes are also sometimes observed	Mature oocytes (> 60%) and a small number of oogonia	2.5
4. Spent stage	V	Ovarian tissue is thin. Connective tissue, hemocytes, and yellow-white granules are markedly observed. Ovarian follicles are unclear. Different stages of oocytes and scattered yolk globules are observed. Phagocytosis is often observed	Oogonia and oocytes are at different stages	0

At the reference site (Hiraiso), the female maturation score of *T. clavigera* specimens decreased sharply during August and September 2017 from a peak in June 2017 (Fig. 2a), and a gradual increase was observed from February to May 2018. The peak female maturation score in 2018 was observed in July, and then it sharply decreased from August to September 2018. A gradual recovery of the female maturation score was observed from February to May 2019. On the basis of the ovarian developmental grade composition and the female maturation scores of the *T. clavigera* specimens collected at Hiraiso, we judged the mature period of *T. clavigera* at Hiraiso to be June and July of each year. We judged October to February to be the non-reproductive season of *T. clavigera* at Hiraiso, because immature ovaries (grade I/recovery stage) were predominantly observed in the *T. clavigera* specimens collected in those months.

At Tomioka, the female maturation score of *T. clavigera* specimens peaked from May to July 2017 and then decreased sharply in August and September 2017 (Fig. 2b). Similar to the trend at Hiraiso, the score at Tomioka increased again from February to May 2018. The peak female maturation score in 2018 was observed in May and June, and a sharp decrease was observed in July. Although a brief increase was observed in August 2018, the score then decreased again until October. The female maturation score was zero from October 2018 to January 2019, but it rapidly recovered from February to March 2019. On the basis of the ovarian developmental grade composition and female maturation scores of the *T. clavigera* specimens collected at Tomioka, we judged their mature period to be May to June or July and their non-reproductive season to be from September to February, when grade V (spent stage) ovaries were predominantly observed.

**Figure 3:**
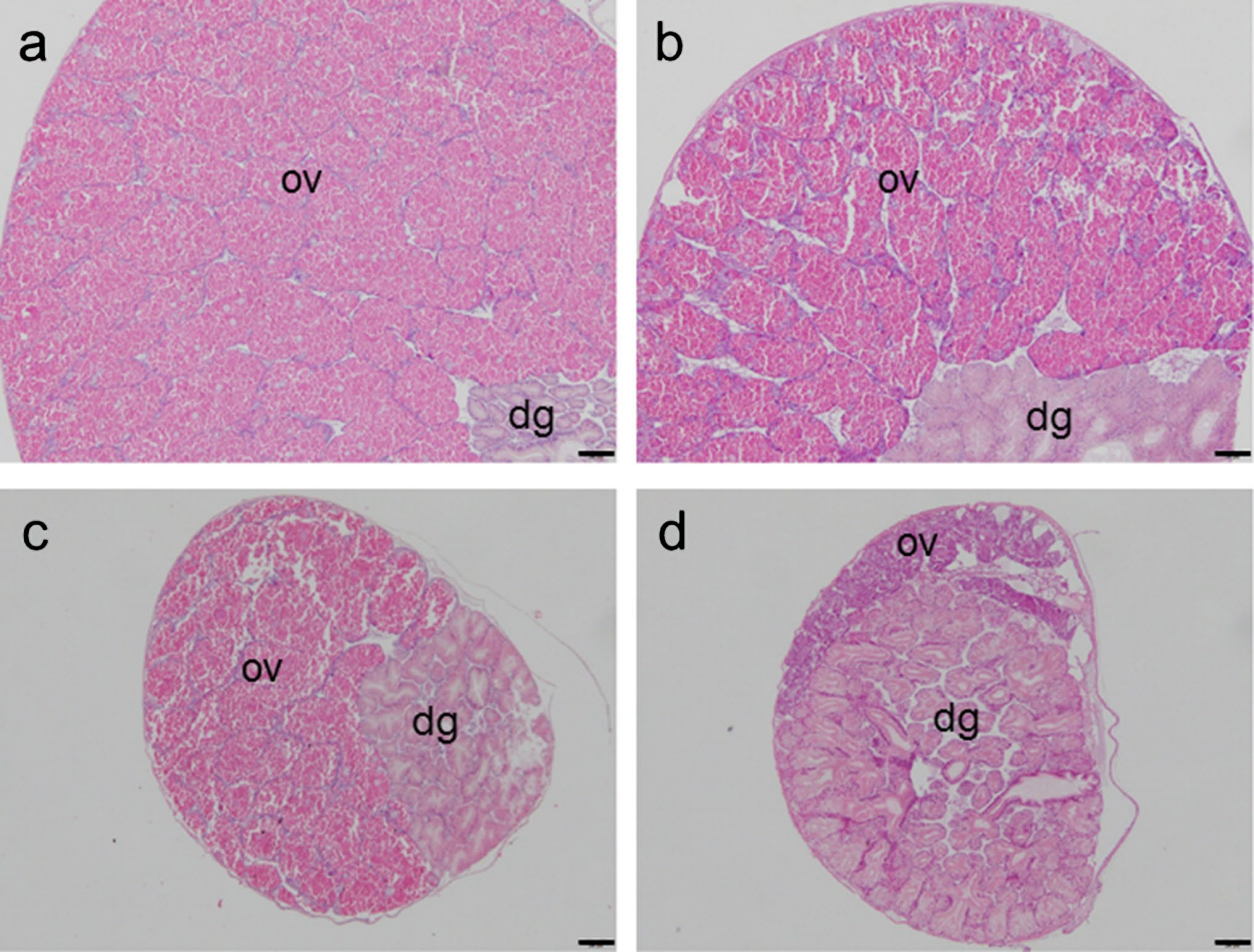
Examples of ovarian tissues of *Thais clavigera* collected at Okuma and Hiraiso. (**a**) Female *T. clavigera* collected at Okuma on 16 June 2018 in the reproductive season, (**b**) female *T. clavigera* collected at Okuma on 24 January 2019 in the non-reproductive season, (**c**) female *T. clavigera* collected at Hiraiso on 18 June 2018 in the reproductive season, and (**d**) female *T. clavigera* collected at Hiraiso on 25 January 2019 in the non-reproductive season. ov, ovary; dg, digestive gland. Bars = 200 µm. (**a**)–(**c**) Ovarian tissues are thick, and many mature oocytes can be seen. Therefore, we judged these ovaries to be mature (developmental grade III; see Table 1). (**d**) The ovarian tissue is thin, and oogonia and early stage oocytes are present, together with connective tissues and hemocytes. Therefore, we judged this ovary to be in the recovery stage (developmental grade I; see Table 1).

At Okuma, the female maturation scores of *T. clavigera* specimens were at peak levels during April–September 2017. Thereafter, female maturation scores remained high (> 2.5) throughout most of the survey period. Moreover, the percentage occurrence of mature ovaries (i.e., grades III and IV/mature stage) was high at Okuma during most of the survey period. Thus, the reproductive cycles of female *T. clavigera* specimens differed markedly at Okuma compared to those at Hiraiso and Tomioka (Fig. 2c). In addition, in *T. clavigera* specimens collected at Okuma and Hiraiso in the reproductive and non-reproductive seasons (June 2018 and January 2019, respectively; Fig. 3), maturation was clearly observed in ovarian tissues of the specimens collected at Okuma in both June 2018 and January 2019, whereas it was observed in only one female specimen collected at Hiraiso in June 2018. Therefore, consecutive sexual maturation apparently occurred in female *T. clavigera* at Okuma, the site closest to the FDNPP.

At Okuma, we also collected specimens of *T. bronni*, a *T. clavigera* relative, and examined the female reproductive cycle, mature period, and other reproductive characteristics for comparison with the female *T. clavigera* specimens collected at the same site (Fig. 2d). Although the female maturation scores of the *T. bronni* specimens showed a reproductive cycle, high (> 2.5) female maturation scores and a high percentage occurrence of mature ovaries (i.e., grades III and IV/mature stage) were observed for much longer periods compared with female *T. clavigera* specimens at Hiraiso and Tomioka. Thus, though the tendency was not as marked as in female *T. clavigera*, consecutive sexual maturation was also observed in female *T. bronni* specimens collected at Okuma.

### Reproductive cycles and sexual maturation in male *T. clavigera* and *T. bronni*

**Figure 4:**
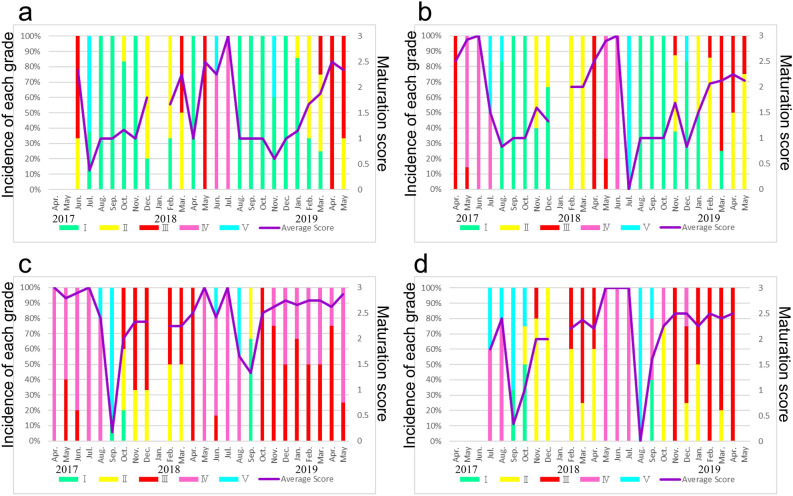
Time series of maturation scores and development grades of male *Thais clavigera* and *Thais bronni* collected at (**a**) Hiraiso (*T. clavigera*), (**b**) Tomioka (*T. clavigera*), (**c**) Okuma (*T. clavigera*), and (**d**) Okuma (*T. bronni*).

The temporal changes in the average monthly maturation scores of testes of *T. clavigera* and *T. bronni* specimens represent the male reproductive cycle (Fig. 4). Developmental grades III and IV (corresponding to maturation scores 2.5 and 3, respectively) were recognized as sexually maturity in testes of *T. clavigera* and *T. bronni* (Fig. 4 and Table 2).

**Table 2:** Stages, developmental grades, and maturation scores of testes of *Thais clavigera* and *Thais bronni*.

Stage	Developmental grade	Characteristics of testicular tissue	Primary cells observed in the testis	Maturation score
1. Recovery stage	I	Testicular tissue is thin. Connective tissue, hemocytes, and yellow-white granules are observed. Spermatogonia and a small number of spermatocytes are observed in the testicular vesicle	Spermatogonia and spermatocytes	1
2. Active stage	II	Testicular tissue is still thin. The number of spermatocytes is increasing, and a small number of spermatids and spermatozoa are sometimes observed in the testicular vesicle	Spermatocytes and a few spermatogonia, spermatids, and spermatozoa	2
3. Mature stage	III	Testicular tissue is becoming thick, and spermatogenesis is proceeding. Various kinds of sperm cells (spermatogonia, spermatocytes, spermatids, and spermatozoa) are observed in testicular tissue	Spermatocytes, and also spermatids and spermatozoa	2.5
	IV	Testicular tissue is thick (approximately > 50% of the cross section of testis and digestive gland). Although various kinds of sperm cells (spermatogonia, spermatocytes, spermatids, and spermatozoa) are observed in testicular tissue, the number of spermatids and spermatozoa is markedly increasing. Many unidentified cells that are almost the same size as spermatocytes and have many granules intensively stained by hematoxylin are also observed	Spermatids and spermatozoa account for > 25% of cells in testicular tissue	3
4. Spent stage	V	Testicular tissue is thin. As in thick testicular tissue, marked gaps or intercellular spaces are observed in the testicular vesicle. Unreleased spermatozoa and unidentified cells, which are almost the same size as spermatocytes and have many granules intensively stained by hematoxylin, are also sometimes observed	Unreleased spermatozoa, and a small number of spermatogonia and spermatocytes	0

At Hiraiso, the male maturation score of *T. clavigera* specimens decreased sharply in July 2017 from a peak in June (Fig. 4a). Subsequently, it gradually increased to another peak in July 2018, which was followed by a sharp decrease in August. Thus, the trend in 2018 was similar to that in 2017. The male maturation score gradually recovered from January to May 2019. On the basis of the testicular developmental grades of the testicular tissue and the male maturation scores, we judged the mature period of male *T. clavigera* at Hiraiso to be June and July. In the non-reproductive season, from August to November or December, the testes of most specimens were immature (grade I/recovery stage).

At Tomioka, the male maturation score of *T. clavigera* specimens peaked in May–June 2017 and then decreased sharply in July and August (Fig. 4b). It increased thereafter from November 2017 to April 2018, in a similar pattern to that at Hiraiso. In 2018, the peak male maturation score was observed in May and June, and a sharp decrease was observed in July. A gradual recovery was observed from November 2018 to May 2019. On the basis of the testicular developmental grades and the male maturation scores, we judged their mature period to be April–June (but not, perhaps, in 2019). In the non-reproductive season, from August to October, immature testes (grade I/recovery stage) were predominantly observed.

At Okuma, the male maturation score of *T. clavigera* specimens was at peak levels during April–July 2017, and it decreased sharply in September 2017 (Fig. 4c). Thereafter, male maturation scores remained high (≥ 2.5), and a high percentage occurrence of mature testes (i.e., mature stage/grades III and IV), were observed throughout most of the survey period (except in August and September 2018). Thus, the male reproductive cycle differed markedly between specimens collected at Okuma and those collected at Hiraiso and Tomioka. Therefore, we also observed apparently consecutive sexual maturation in male *T. clavigera* specimens collected at Okuma, although the pattern appeared somewhat weaker than that in female *T. clavigera* collected there.

We also compared the reproductive cycle, mature period, and other male reproductive characteristics in male *T. bronni* specimens collected at Okuma with those of the male *T. clavigera* specimens collected at the same site (Fig. 4d). Although the male maturation score of *T. bronni* specimens collected at Okuma showed a reproductive cycle (characterized by an abrupt decline of the male maturation score in September 2017 and August 2018, similar to the declines observed in male *T. clavigera* specimens collected at Okuma), high (≥ 2.5) male maturation scores and a high percentage occurrence of mature testes (i.e., grades III and IV/mature stage) were observed over a much longer period at Okuma than at Hiraiso and Tomioka, Therefore, we also observed apparently consecutive sexual maturation in male *T. bronni* at Okuma, although, similar to female *T. bronni* compared with female *T. clavigera*, the tendency was less marked in male *T. bronni* than in male *T. clavigera*.

## Discussion

We observed differences in shell size (i.e., shell height and weight) of *T. clavigera* specimens among the three sites; in general, specimens collected at Okuma were bigger than those collected at Hiraiso and Tomioka. Because population densities (i.e., abundances) of mussels (*Mytilus galloprovincialis* and *Septifer virgatus*) and barnacles (*Chthamalus challengeri*), which are prey for *T. clavigera*, were higher at Okuma than at Tomioka and Hiraiso^14^, the growth rate of the *T. clavigera* population might have been faster at Okuma than at Tomioka and Hiraiso. However, because the shell height of all of the studied *T. clavigera* specimens collected at Okuma, Tomioka, and Hiraiso exceeded 12 mm, the minimum shell height of a mature specimen^21^ (Supplementary Table S1), the reproductive cycle and sexual maturation in the female and male *T. clavigera* specimens could be compared among the sites.

We inferred that the breeding season of the *T. clavigera* population at Hiraiso during the summer, on the basis of the observed female and male reproductive cycles (Figs. 2a and 4a). Kon et al.^22^ reported that the breeding season of the *T. clavigera* population at Yoriihama, Niigata, Japan, extended from late spring to summer. In general, *T. clavigera* populations in Japan mature from late spring to summer, and the females deposit their egg capsules beneath ledges or on other substrates (e.g., tetrapods and similar concrete structures set along the coast for wave protection)^23^. At Okuma, however, we observed sexual maturation in both female and male *T. clavigera* specimens throughout most of each year of the survey period (April 2017 to May 2019) (Figs. 2c and 4c). Moreover, we observed consecutive sexual maturation in both female and male *T. bronni* specimens collected at Okuma, though the tendency was less marked than it was in *T. clavigera* (Figs. 2d and 4d). Although we observe a rapid regression of the testis in male *T. clavigera* specimens collected at Okuma in September of both 2017 and 2018, they recovered rapidly to maturation in October of both years (Fig. 4c). Similar to *T. clavigera*, we also observed a rapid regression of the testis in male *T. bronni* specimens collected at Okuma in September 2017 and August 2018, followed by recovery to maturation 2 months later, in November 2017 and October 2018, respectively (Fig. 4d). Therefore, both *T. bronni* and *T. clavigera* populations apparently breed throughout most of the year at Okuma. In fact, we observed egg laying (i.e., egg capsules deposited on substrates) by *T. clavigera* and *T. bronni* at Okuma as late as 21 September in 2017 and 10 September in 2018. Unfortunately, it is impossible to directly observe egg laying by *T. clavigera* and *T. bronni* on substrates at Okuma and other sites from October to next March because the tide level is high, even at low tide, and wave action is strong. To the best of our knowledge, consecutive sexual maturation and egg laying in mid-September have not been reported previously in *T. clavigera* and *T. bronni*.

It is possible that, with respect to their reproductive cycle, the *T. clavigera* population at Tomioka might be intermediate between Okuma and Hiraiso, because we observed spent ovaries (grade V/spent stage) in female *T. clavigera* specimens predominantly from September to February at Tomioka, whereas at Hiraiso, we observed immature ovaries (grade I/recovery stage) predominantly from October to February (Fig. 2b). Thus, the non-reproductive period differed slightly between Tomioka and Hiraiso.

Was the consecutive sexual maturation observed in the *T. clavigera* and *T. bronni* populations at Okuma related to radionuclides leaked from the FDNPP? First, the *T. clavigera* and *T. bronni* specimens collected at Okuma in the present study did not seem to be the direct offspring of parent shells that had probably been exposed to radionuclides leaked during the March 2011 nuclear disaster because no rock shell (*T. clavigera*) specimens were collected near the plant, from Hirono to Futaba Beach (a distance of approximately 30 km) in April 2012^13^. The absence of rock shells at sites close to the FDNPP in April 2012 suggests the likely mortality of almost all individuals living there after March 2011 and, therefore, that reproduction and recruitment either did not occur there or were less successful in summer and autumn (the reproductive season and thereafter) of 2011. However, why adult rock shells living near the FDNPP disappeared or had little or no reproductive success in 2011 is still unknown^13^. Second, the *T. clavigera* and *T. bronni* specimens collected at Okuma in the present study seemed to be offspring of parent shells that had inhabited various sites to the north and south of the FDNPP^14^. The early life stage of *T. clavigera* and *T. bronni*, which lasts more than 2 months^21^, is a free-swimming planktonic veliger. Therefore, the *T. clavigera* and *T. bronni* specimens collected at Okuma might have been spawned in remote areas and have subsequently traveled as veligers to the intertidal zone at Okuma through dispersion and migration processes. In addition, the activity concentrations of radionuclides, including radiocesium (^134^Cs and ^137^Cs), in seawater have been mostly less than 0.1 Bq/L, even at sites close to the FDNPP, since April 2017 (https://radioactivity.nsr.go.jp/ja/contents/9000/8141/24/engan.pdf; in Japanese). Therefore, the dose rates of the *T. clavigera* and *T. bronni* specimens collected at Okuma in the present study could be much lower than 400 μGy/h (= 9.6 mGy/d), which is the threshold value estimated by the United Nations Scientific Committee on the Effects of Atomic Radiation for a detrimental effect on populations of aquatic organisms^24^. Thus, it appears to be unlikely that the consecutive sexual maturation observed in both *T. clavigera* and *T. bronni* populations at Okuma in the vicinity of the FDNPP is associated with radionuclides such as ^137^Cs leaked from the power plant.

In general, the reproductive cycle (including spawning behavior) of gastropods, including *Thais* spp., is controlled by both external (i.e., water temperature, illumination/photoperiod, daily and monthly tidal cycles, and physical factors such as rough seas and onshore winds) and internal factors (i.e., genetics, nutrition, and neurohormones/neuropeptides)^25–27^. In addition, at low latitudes, an extended reproductive season is common, whereas at high latitudes the season tends to be restricted by the need for the phytotrophic veliger stage to coincide with phytoplankton blooms. Variation in the length of the reproductive season within their geographic range is evident in two species, *Hinia incrassata* and *Lacuna vincta*, both with planktonic veligers, that occur at Plymouth, U.K. The former, whose range extends into warmer waters, breeds during spring and summer at Plymouth but throughout the year in Bermuda^28^, whereas the latter, whose range extends into colder waters, breeds during January–May at Plymouth and in June–July or August in the White Sea, an inlet of the Barents Sea^29^. Yamaguchi^30^ reported that the green snail (*Turbo marmoratus*), which has separate sexes, is able to reproduce at the age of about 4 years when its shell width reaches about 130 to 150 mm. At higher latitudes, however, this snail appears to breed only in the summer months, when the water temperature is higher, whereas at lower latitudes, mature individuals breed repeatedly throughout the year^30^. In addition, giant clam (*Tridacna gigas*) populations reproduce throughout the year at the low-latitude site of Palau^31^.

Different from the high water temperatures observed year-round in tropical and subtropical areas, water temperatures at Onahama, Iwaki city (on the southern Fukushima coast) fluctuated from 8.4 to 25.2 °C in 2017, from 7.0 to 24.2 °C in 2018, and 9.7 to 24.9 °C in 2019 (https://www.pref.fukushima.lg.jp/uploaded/attachment/363615.pdf; in Japanese), whereas those at Matsukawa-ura, Soma city (on the northern Fukushima coast), fluctuated from 6.1 to 26.1 °C in 2017, from 5.0 to 28.5 °C in 2018, and from 7.0 to 27.2 °C in 2019 (https://www.pref.fukushima.lg.jp/uploaded/attachment/363616.pdf; in Japanese) (Fig. 1). Thus, it is unlikely that the consecutive sexual maturation observed in both female and male *T. bronni* and *T. clavigera* specimens collected at Okuma in the present study was dependent on high water temperatures. Although we observed egg laying (i.e., egg capsules laid on substrates) by both *T. clavigera* and *T. bronni* at Okuma in September of both 2017 and 2018, to the best of our knowledge, consecutive sexual maturation and egg laying in mid-September by *T. clavigera* and *T. bronni* has not been reported previously. Thus, both these phenomena are unusual and differ markedly from our observations at Hiraiso and the findings reported by Kon et al.^22^ for the *T. clavigera* population in Niigata, Japan.

We observed consecutive sexual maturation in both sexes of *T. clavigera* and *T. bronni* specimens collected at Okuma over about 2 years (April 2017 to May 2019; Figs. 2c, 2d, 4c, and 4d). Thus, rather than a temporary phenomenon, it is probably a site-specific phenotypic response, possibly caused by specific environmental factors near the FDNPP.

We speculate that consecutive sexual maturation may be caused by either a maturation switch being turned on all the time, or that it is an irreversible response such that once maturation starts, a return to the original physiological phase is not possible. The endocrinology and reproductive physiology, such as controls for sexual maturation, of gastropods, especially prosobranchs, are not yet fully understood. It has been suggested that gastropods might have two co-existing regulatory systems for reproduction: neurohormones/neuropeptides and vertebrate-type steroid hormones^32^. Recently, however, doubts have been raised as to whether gastropods inherently have vertebrate-type steroids that function as sex hormones^33–37^. Thus, in the future, a comparative endocrinology study should compare expression levels of neuropeptides and their seasonal/temporal variations between the *T. clavigera* population at Okuma and that at Hiraiso to elucidate the mechanism of consecutive sexual maturation in the Okuma population.

The possibility that nuclear receptors, such as the retinoid X receptor (RXR), are involved in the development of consecutive sexual maturation in *T. clavigera* and *T. bronni* at Okuma should also be investigated. RXR is known to be involved in the induction of imposex, a type of endocrine disruption, in gastropods^38–45^. Imposex is an irreversible syndrome in which male sexual characteristics and organs such as the penis and vas deferens are superimposed in female prosobranch gastropods. Very low concentrations (ppt or ng/L) of organotin compounds such as tributyltin and triphenyltin, which are used in antifouling paints for ships and fishing nets, are known to induce imposex in prosobranch gastropods^46–53^. In severe cases, imposex may result in a sex change (i.e., spermatogenesis in the ovary, or a change of ovarian tissue to testicular tissue)^54–57^. Thus, RXR may also have a physiological effect of inducing gonad abnormalities, including the consecutive sexual maturation observed in the *T. clavigera* and *T. bronni* specimens collected at Okuma.

Rock shells (*T. clavigera*, and *T. bronni*) have a free-swimming planktonic veliger early life stage that lasts more than 2 months^21^. Therefore, the *T. clavigera* and *T. bronni* specimens collected at Okuma could have originated in remote areas and have traveled to the intertidal zone at Okuma as veligers through dispersion and migration processes. Therefore, it is unlikely that consecutive sexual maturation is congenital in the *T. clavigera* and *T. bronni* specimens collected at Okuma. Instead, exposure of *T. clavigera* and *T. bronni* larvae in the (late) planktonic stage or in the juvenile stage after settlement in the intertidal zone at Okuma to certain environmental factors around the FDNPP may have triggered the induction of consecutive sexual maturation in the Okuma populations.

Changes in life history traits and population dynamics of marine organisms in response to environmental change are known to occur in nature, although the causes underlying the responses remain unidentified^58^. Kodama et al.^59^ reported changes in the growth and reproductive traits of the dragonet, *Callionymus valenciennei*, in Tokyo Bay, Japan, that coincided in a decrease in stock size. The minimum standard length at which the dragonet attains gonadal maturation was smaller in the 2000s (4.8 cm) than in the 1990s (6.0 cm). In addition, the timing of the onset of spawning was earlier in the 2000s (spring) than in the 1990s (summer). Kodama et al.^59^ also found significant changes in the growth in both sexes from the 1990s to the 2000s; growth after sexual maturity was attained was significantly decreased in the 2000s compared with the 1990s. Changes in life history traits may reflect a trade-off in the allocation of available energy resources between reproduction and somatic growth; under limited prey abundance, more energy resources may be allocated to reproduction to enhance stock recovery. These findings suggest that consecutive sexual maturation in *T. clavigera* and *T. bronni* populations at Okuma, where no individuals of these species were observed from December 2011 to July 2016, following the FDNPP accident^13,14^, may reflect such a trade-off to enhance the recovery of the populations, although we have no data indicating that such an energy resource allocation trade-off has occurred. It is also unclear whether this life history change (i.e., consecutive sexual maturation) might, for example, cause the rock shell populations to be more resilient to the negative effects of low water temperature on larval and juvenile survival in winter. Thus, the impacts of consecutive sexual maturation on *T. clavigera* and *T. bronni* populations are unknown. Future studies in ecotoxicology as well as in the developmental biology and ecology of the early life stages of these species are needed to identify the causal factors of consecutive sexual maturation and to evaluate its effects on the population dynamics of *T. clavigera* and *T. bronni* at Okuma.

Further field studies are also necessary to constrain the geographic area in which consecutive sexual maturation in *T. clavigera* specimens occurs, and whether other species in the intertidal zone at Okuma also exhibit consecutive sexual maturation.

## Methods

### Collection of rock shell specimens

From April 2017 to May 2019, we collected specimens of rock shells monthly at two sites in Fukushima prefecture near the FDNPP. At Ottozawa (Okuma town, ca. 1 km south of the FDNPP), we collected both *T. clavigera* and *T. bronni* (currently, recognized as *Reishia clavigera* (Küster, 1860) and *Reishia bronni* (Dunker, 1860), respectively; Gastropoda, Neogastropoda, Muricidae) specimens, and at Tomioka fishing port (Tomioka town, ca. 10 km south of the FDNPP), we collected *T. clavigera* specimens. We also collected *T. clavigera* specimens at a reference site in Ibaraki prefecture (Hiraiso, Hitachi-naka city, ca. 120 km south of the FDNPP). On each collection date, we collected 7–14 individuals, on average 10.3 *T. clavigera* and 10.0 *T. bronni* individuals (Fig. 1, Supplementary Table S1). After collection, the specimens were dissected and their sex was determined by examining their accessory sex organs^60^. Then, the specimens were fixed in Bouin’s fluid (see below) for 48 h for later histological examination of the gonads.

### Histopathological examination of *T. clavigera* and *T. bronni* gonads

The gonads of the rock shell specimens collected monthly at each site were fixed in Bouin’s fluid (saturated picric acid : formaldehyde solution : acetic acid = 15 : 5 : 1, all reagents were guaranteed to be extra pure by Nacalai Tesque, Inc.) for 48 h, embedded in paraffin (Sigma-Aldrich, Paraplast Plus), cut into 5–6 μm sections with a rotary microtome (Leica, RM2125RT), and stained with hematoxylin (Tissue-Tek Hematoxylin 3G) and eosin (Tissue-Tek Eosin) by an autostaining machine (Leica, Autostainer XL). Histopathological examination of the gonads was conducted under a light microscope (Olympus, BX40) equipped with a digital camera (Olympus, DP22), and the images were analyzed by using image analysis software (Olympus, cellSens Standard V2.2 (Build 17,989) (https://www.olympus-lifescience.com/en/software/cellsens/#!cms[focus]=cmsContent6017)) installed on a personal computer (Hewlett-Packard, DP2-PC-S-1–21).

**Figure 5:**
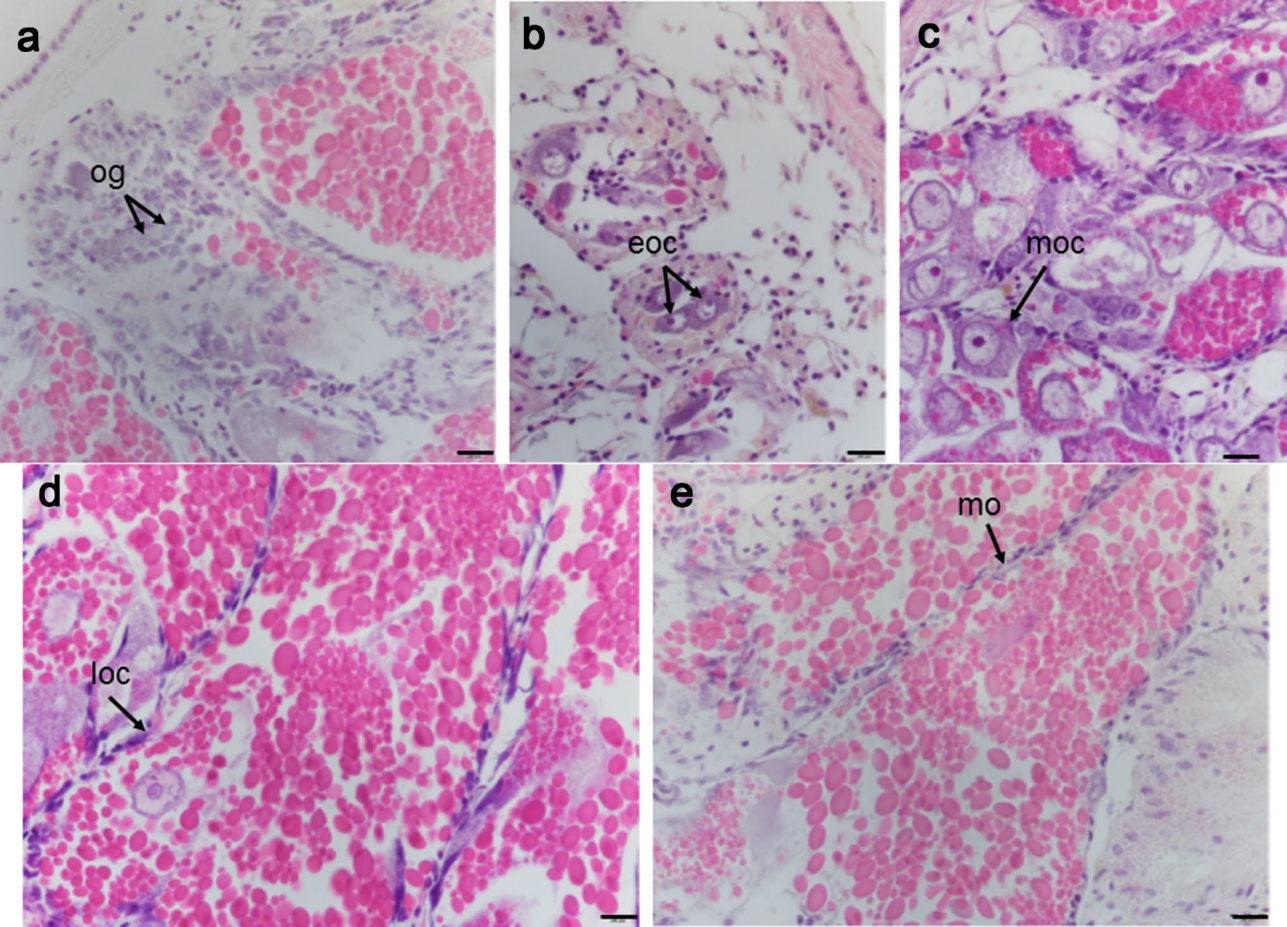
Reproductive cells in ovaries of *Thais clavigera*: (**a**) oogonium (og), (**b**) early stage oocyte (eoc), (**c**) middle stage oocyte (moc), (**d**) late stage oocyte (loc), and (**e**) mature oocyte (mo). Bars = 200 µm.

To quantitatively evaluate gonadal maturation in the rock shell specimens, female and male reproductive cells were scored according to a developmental grading system similar to that described by Takamaru and Fuji^61^. Female reproductive cells were categorized according to their degree of maturity as an (1) oogonium or an (2) early (eoc), (3) middle (moc), (4) late (loc), or (5) mature (mo) oocyte (Fig. 5). An oogonium has a nucleus containing chromatin chains and a small nucleolus, as well as small, hematoxylin-stained granules. The cytoplasm is thin and its stainability is weak (Fig. 5a). An early stage oocyte is surrounded by a single layer of follicular cells. The large nucleus contains dispersed or scattered chromatin and a large basophilic nucleolus. The early stage oocyte is larger than an oogonium, and it has an irregular shape (Fig. 5b). No yolk globule accumulation is observed in either the oogonium or the early stage oocyte. In the middle stage, the oocyte starts to accumulate eosinophilic yolk granules in the cytoplasm. Its nucleolus is large and basophilic, and chromatin is dispersed in the nucleus. The middle stage oocyte is larger than an early stage oocyte and contains a few yolk globules stained by eosin (Fig. 5c). The late stage oocyte accumulates more eosinophilic yolk globules in the cytoplasm. Its outline is becoming unclear, and its nucleolus is more weakly stained. The late stage oocyte is larger than the middle stage oocyte and contains several yolk globules stained by eosin (Fig. 5d). A mature oocyte has an unclear outline, and its cytoplasm is filled with yolk globules. Its nucleus has an indeterminate form, and the nuclear membrane has disappeared. Small yolk granules cluster around the nucleus (Fig. 5e).

The developmental grade of an ovary could range from I to V and was determined on the basis of the characteristics of the ovarian tissue and the primary cells observed in the ovary (Table 1). Then, a maturation score based on the developmental grade of the ovary was assigned to each female *T. clavigera* and *T. bronni* specimen collected monthly at each site (Table 1).

**Figure 6:**
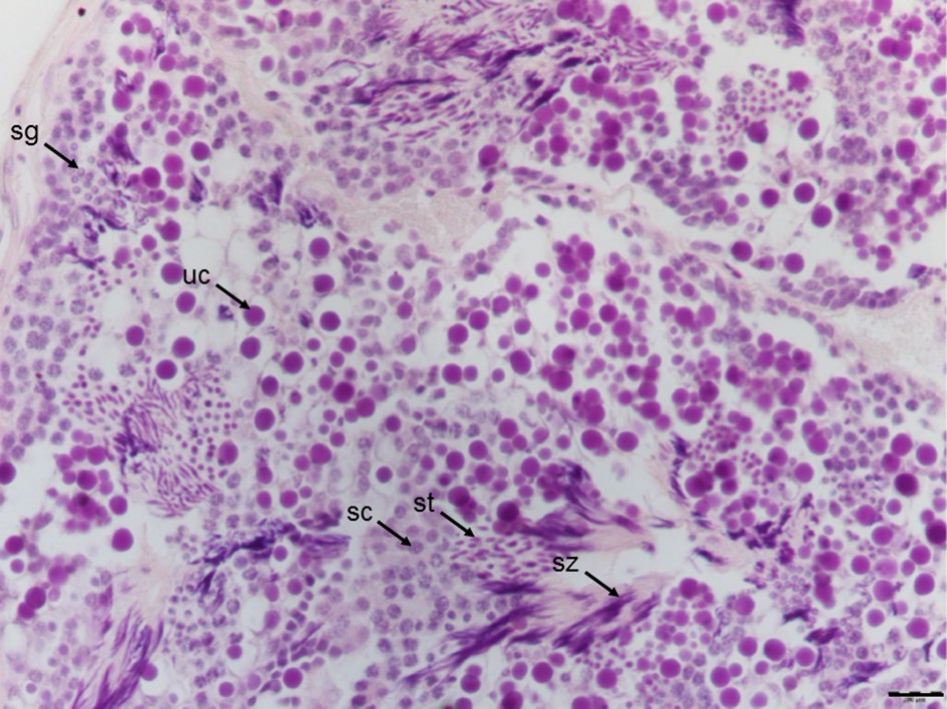
Reproductive cells in a testis of *Thais clavigera*: spermatogonium (sg), spermatocyte (sc), spermatid (st), and spermatozoon (sz). Unidentified cells (uc) that were almost the same size as spermatocytes and contained many granules intensively stained by hematoxylin were observed in the testes of both *Thais bronni* and *T. clavigera*. Bar = 200 µm.

Male reproductive cells were also categorized according their degree of maturity as a (1) spermatogonium, (2) spermatocyte, (3) spermatid, or (4) spermatozoon (Fig. 6). The spermatogonium, which is about half the size of a spermatocyte, is an almost round cell having a nucleus that contains small granules stained by hematoxylin. The stainability of the cytoplasm is weak. A spermatocyte is a round cell with a large nucleus and scattered punctiform chromatin. The stainability of a spermatocyte is weak. The spermatid is generally an oval or round cell that is intensively stained by hematoxylin, but some spermatids are crescent- or rod-shaped, with a reduced volume of cytoplasm. The spermatozoon is a thread-shaped cell that is intensively stained by hematoxylin. Some cells almost the same size as spermatocytes but with many granules intensively stained by hematoxylin were also observed in the testes of *T. clavigera* and *T. bronni*.

The developmental grade of a testis, ranging from I to V, was determined from the characteristics of the testicular tissue and the primary cells observed in the testis (Table 2). Then, a maturation score based on the developmental grade of the testis was assigned to each male *T. clavigera* and *T. bronni* specimen collected monthly at each site (Table 2).

Average maturation scores of ovaries and testes of *T. clavigera* and *T. bronni* specimens collected each month at each site were calculated. The temporal changes of these scores were considered to represent the reproductive cycle in each species^61^.

### Data availability

All data generated or analyzed in this study are included in this published article or in the Supplementary Information.

## Supplementary Information

Supplementary Table
